# Evaluation of a brief pilot psychoeducational support group intervention for family caregivers of cancer patients: a quasi-experimental mixed-methods study

**DOI:** 10.1186/s12955-017-0595-y

**Published:** 2017-01-23

**Authors:** Rathi Mahendran, Haikel A. Lim, Joyce Y. S. Tan, Hui Ying Ng, Joanne Chua, Siew Eng Lim, Ee Heok Kua, Konstadina Griva

**Affiliations:** 1 Department of Psychological Medicine, National University of Singapore, NUHS Tower Block, Level 9, 1E Kent Ridge Road, Singapore, 119228 Singapore; 2 Duke-NUS Medical School, 8 College Road, Singapore, 169857 Singapore; 3 Department of Psychology, National University of Singapore, Block AS4 #02-07, 9 Arts Link, Singapore, 117570 Singapore; 4 Department of Haematology-Oncology, National University Cancer Institute, Singapore, NUHS Tower Block, Level 7, 1E Kent Ridge Road, Singapore, 119228 Singapore; 5 Department of Psychological Medicine, National University Hospital, NUHS Tower Block, Level 9, 1E Kent Ridge Road, Singapore, 119228 Singapore

**Keywords:** Cancer, Oncology, Family caregivers, Quality of life, Psychosocial intervention, Asia

## Abstract

**Background:**

Family caregivers of cancer patients often experience an impaired quality of life (QOL) and emotional distress as a result of their caregiving duties, which may potentially influence the quality of care of their care recipients. The COPE (Caregivers of cancer Outpatients’ Psycho-Education support group therapy) intervention was developed as a response to the lack of work done among family caregivers of ambulatory cancer patients in Asia. This group intervention comprised four weekly sessions simultaneously targeting psychoeducation, skills training, and supportive therapy. The present study sought to evaluate the pilot COPE intervention using both quantitative and qualitative measures. The Hospital Anxiety and Depression Scale (HADS) was used to measure both depression and anxiety, while the Caregiver QOL – Cancer (CQOLC) measured caregiver QOL. These instruments were measured at baseline pre-intervention, and immediately post-intervention. A waitlist control group design was adopted. A subset of caregivers from the intervention group were invited for a semi-structured interview post-intervention.

**Findings:**

Quantitative analyses suggest that while QOL remained stable in control group participants, intervention group participants experienced QOL improvements – both in overall QOL and in the specific domain of burden. There were no significant differences in the trajectories of depression and anxiety in both groups. Qualitative analyses suggest that this might have been a result of the intervention not only equipping participants with the relevant coping skills, but also providing a platform for emotional expression and situational reappraisal.

**Conclusions:**

The COPE intervention has shown some efficacy in helping family caregivers of cancer patients, but more work is required before this can be implemented.

**Trial registration:**

Current Controlled Trials NCT02120183. Registered 17 April 2014. Retrospectively registered.

## Introduction

Providing physical and emotional support to cancer patients [1] often takes a toll on family caregivers [2]. Because caregivers’ quality of life (QOL) and emotional symptomatology may potentially affect the quality of care of cancer patients [3], it is essential to develop supportive care services for caregivers. Psychosocial interventions for caregivers often fall into three categories: psychoeducation, skills training, and therapeutic counseling [4]. While half of existing interventions consider the caregiver–patient unit [5], it has been suggested that individualized caregiver-specific interventions may be more efficacious [6]. Interventions should also be tailored to specific populations; much research on this, however, has been done exclusively in the United States [1].

The COPE (Caregivers of cancer Outpatients’ Psycho-Education support group therapy) intervention was aimed at family caregivers of patients receiving ambulatory cancer care in Singapore. Developed by an interdisciplinary team of psychiatrists, psychologists, and oncology professionals, and based on the principles of Brief Integrative Psychological Therapy (BIPT) [7, 8], it sought to improve caregivers’ QOL by simultaneously targeting psychoeducation, skills training, and supportive therapy. The content of these four weekly sessions were tailored in response to the concerns of caregivers of oncology patients in Singapore [9]. Facilitated by a clinical psychologist, the first 10–15 minutes of each hour-long session was set aside for didactics (i.e., psychoeducation and skills training), while the remaining time was devoted to supportive group therapy. The specific methods of the COPE intervention and trial protocol are described elsewhere [10, 11].

The present study is thus an evaluation of this pilot four-week brief psycho-education support group intervention using a mixed-methods framework, given the nascence of the COPE intervention. The quantitative component focuses on evaluating the intervention in terms of participant outcomes of QOL, depression, and anxiety. The qualitative component (involving interviews) pertains to the process of the intervention, through obtaining participants’ feedback in order to inform improvements in service delivery.

## Methods

### Participants and procedures

This study received ethics approval from the National Healthcare Group Domain-specific Review Board (Reference: 2013/00662) and funding from the National University Cancer Institute, Singapore (NCIS) Seed Fund. Participants were recruited at NCIS outpatient clinics if they met inclusion criteria: (a) between 21 and 74 years; (b) willing to attend hour-long weekly programs for four weeks; (c) able to understand, speak, and read English; (d) a family member living with and providing care and support for the patient. No gender, ethnicity, cancer site or type restrictions were imposed given the exploratory nature of the study. All participants provided written informed consent.

Because we did not want to deny care to participants who were interested and able to attend the intervention, a quasi-experimental design was chosen whereby consenting participants were allocated into two study arms based on their availability to attend the intervention (non-randomized groups). Participants interested and able to attend the next intervention session formed the intervention group. Participants interested but unable to attend the next intervention session were put on the waitlist until they could eventually attend, and formed the control group. However, none of these waitlisted participants were able to attend eventually. Therefore, upon study conclusion, there were 56 participants in the intervention group, and 41 participants in the control group. The demography of the intervention and control groups are presented in Table 1. There were, on average, 6 participants per group.

**Table 1:** Demography of the intervention and control groups

Demographic variables	Intervention (*n* = 56) N	Control (*n* = 41) N
Age (in years)		
21-30	7	8
31-40	12	9
41-50	13	12
51-60	16	9
61-64	6	3
> 64	2	0
Gender		
Male	21	13
Female	35	28
Ethnicity		
Chinese	31	26
Others	25	15
Cancer Type		
Breast	11	11
Nasopharynx/Throat/Oral	3	3
Gyne	4	0
Pancreas	1	5
Blood	11	7
Lung	4	6
Gastrointestinal	12	7
Brain tumour	1	0
Renal	3	2
Prostate	1	0
Multi-site	4	0
Cancer Stage		
Early	13	15
Advanced	41	23
Education		
Primary: Incomplete/PSLE certification	3	3
Secondary: Incomplete/ITE/N/O Levels	15	6
Pre-university: A Levels/Polytechnic Diploma	11	13
University: Undergraduate/Postgraduate degree	27	18
Income^a^		
Less than $2000	12	12
$2000 to $7999	26	15
$8000 and above	10	6
Do not know/prefer not to say	8	8
Relationship with care recipient		
Spouse	21	13
Child	15	17
Others	20	10
Treatment care recipient is currently undergoing/has undergone^b^		
Radiotherapy	11	8
Chemotherapy	41	33
Surgery	10	7
Length of care provided (in months)		
0 to 6	34	16
7 to 12	4	9
13 to 18	4	2
19 to 24	3	1
> 25	11	8

^a^Income brackets follow those specified in the Department of Statistics Singapore. Refer to Key Household Characteristics and Household Income Trends, 2011 http://s3.amazonaws.com/zanran_storage/www.singstat.gov.sg/ContentPages/2546294586.pdf

^b^Treatment type was binary coded as yes/no as care recipient may be receiving/have received more than one type of treatment

**Fig. 1:**
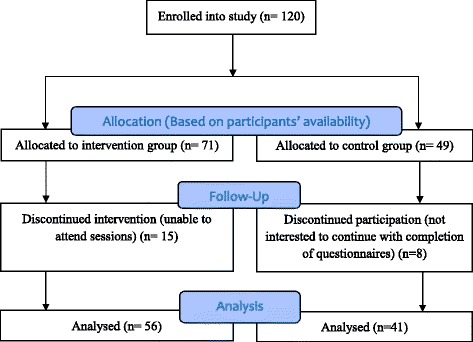
CONSORT diagram outlining the participant flow

Of the participants in the intervention group, 24 (43%) missed at least one session due to their caregiving duties; these participants completed missed sessions in the next run of the intervention. Only 32 (57%) completed the scheduled intervention within four consecutive weeks. The intervention group took an average of 6.06 weeks to complete the program (median = 4; range = 4 to 20). Figure 1 presents the CONSORT diagram for the study.

### Measures

Intervention and control participants completed (a) a sociodemographic questionnaire; (b) Caregiver QOL – Cancer (CQOLC) scale [12], a 35-item instrument on a five-point Likert-type scale (scores ranging 0–140) assessing caregivers’ QOL on the domains of disruptiveness, burden, positive adaptation and financial concerns; and (c) Hospital Anxiety and Depression Scale (HADS) [13], assessing symptoms of depression and anxiety in the past week (maximum score of 21 per anxiety/depression subscale).

Participants completed the questionnaires before the intervention commenced (T1), and immediately post-intervention (T2). A subgroup of 20 participants were invited for an additional 30-minute semi-structured interview at T2 with a research assistant.

### Data analysis

Quantitative analyses were conducted using SPSS 22 (Chicago, IL), with significance levels set at .05. As missing data were infrequent (1%) and completely at random (Little’s MCAR test *p*s > .05), no data imputations were employed [14]. No significant demographic case-mix differences between the intervention (including four-week intervention subgroup) and control groups were found (all chi-square and Fisher’s Exact tests *p*s > .05). As such, to determine if the four-week intervention group improved significantly over the control group, between subjects repeated measures analyses of variance (ANOVAs) were employed. To determine if there were significant differences in baseline scores between the intervention and control groups, independent samples *t* tests were conducted. To determine if there were differences across time in the intervention and control groups, one-way repeated measures ANOVAs were employed.

Qualitative analyses were conducted using NVivo 9 (Doncaster, Australia), with post-intervention interviews transcribed and coded independently by two individuals. An inductive, data-driven approach was first used to identify codes arising in the dataset [15], following which iterative coding and constant comparison [16] was used to develop a framework of codes to describe the data. These were reviewed amongst three coders (agreement α = .85) until a consensus was achieved.

## Results

### Quantitative analyses

**Fig. 2:**
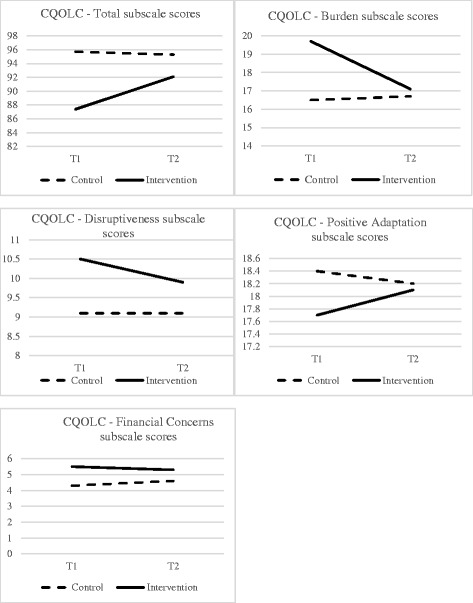
Change in CQOLC scores over time for the control and intervention groups

There were no significant interactions between the intervention and control groups on all measures of interest (all *p*s > .05). At baseline, intervention group participants reported impaired QOL and greater depressive and anxious symptoms than control group participants (*p*s < .05). QOL, depression, and anxiety remained stable in the control group across time (*p*s > .40), but there were significant post-intervention improvements for intervention participants in overall QOL (*p* = .053), and specifically in the domain of burden (*p* = .034). Figure 2 graphically presents the QOL total and domain scores for both the intervention and control groups.

### Qualitative analyses

Five main themes surfaced from the post-intervention interviews. Participants highlighted the importance of group processes in creating a central pool of knowledge and a safe space that would allow them to diffuse their emotional turmoil through opportunities for self-expression. Concrete gains were achieved through normalization of such caregiving experiences and the experiential learning of coping skills, which they perceived to have also benefited their care recipients. The group also allowed participants to reflect and reappraise their own behavior and situations through making comparisons with other caregivers. Facilitation by the psychologist was helpful; psychoeducation and skills training were not only informative, but also validated their experiences and feelings. Finally, there was feedback that the support group format could be modified to include the medical aspect of cancer care and accommodate caregivers’ schedules, through employing other media forms such as an online platform or informal meetings. Table 2 presents participants’ quotes from the interviews, and the themes.

**Table 2:** Themes emerging from the qualitative interviews and the supporting quotes

Themes that emerged	Extracted quotes
Support group as providing knowledge and a safe space for self-expression	Providing information and third person view of situation, and triggering realisation of personal involvement in building a knowledge community: It has helped me to have a better realization. Other people are going through the same things, the caregivers, and maybe this is making me more aware of things that have been happening in my own situation, and it has broaden up and help me to realize, make me more aware of it, whereas I might have just sort of say “well this is going on but I am not sure what I am going to do but I will just file it away.” It helps to bring our things like that; we talk about that and we share that . . . it helps me to share and help me to realize that it is beneficial for me to share these personal things of my own because other people may be going through them and maybe afraid to talk about or share them. As a safe space: You see her [referring to another caregiver], outside she won’t talk. She only chat about everyday thing. Inside, she will talk so much. It’s a healthy talk. At least somebody listens to me. Normally people won’t listen to you, but in this group you can talk and people will give you feedback, advice.
Normalization of experiences	Learning about illness-related, caregiving and lifestyle norms through the group. . . . if everyone in the group is same, then I’ll know if it’s a norm, or if people say, “no it’s not a norm, [the patient] shouldn’t be complaining of this”, then I would know. In observing how others handled the caregiving role and reappraising their situation: [Another caregiver] is like, how to say, I learnt a lot from her. It really made me see. The first time you see me I was really frustrated, “whether I am just going here, or am I just wasting my time?” Like there is no purpose. But after seeing like [the caregiver], she was feeding her husband, through the tube . . . She take [it] all [in] stride, even though she was tired, she really looked after him. She maybe, I don’t know how to say, [she is] sincere. [These caregivers] are taking it like [it’s] nothing. . . . Right now I know, after all these sessions, what I want to do. It gave me a clearer view on how to manage my mum’s actions and my son’s too. I feel that I don’t really 100% love him. Maybe I only do 70%, not enough, the love that I give is not strong enough, not like those people [in the support group] who are sharing. . . . [Now I am] not so harsh lah, when my husband tells me, then I just keep quiet. Last time I used to answer back but now I [just say] ‘hm’. As a form of reassurance: it helps reassure the caregiver that he/she is doing the max. . . . I was afraid that my caregiving role wasn’t so well done, but the support group shows that I’m coping so it makes me a bit relieved. It gives me a checklist of the things I should be doing, so I can make sure that I’m not deviating too much.
Experiential learning of coping skills	. . . you can gather these [visualization skills] from books. With this support group I think the difference is that I experience [it] myself, so I think it makes a difference. I learn what she had shared. I personally experience the technique and they are helpful for me. I think on the third session onwards I start to talk to this Malay lady [other caregiver’s name] and today it is like we can share freely. I feel that it will be good if we can share freely. It is like [the psychologist] is being a trainer to us, but between us we are able to share our experiences and interact in that way.
Challenging negative cognitions	Reframing cognitions of caregiving situation: In the past it used to be “not happy”, when I ask her questions she will keep quiet until I press on, like “eh I’m talking to you”. And she’ll answer reluctantly and negatively. Like the “don’t ask me, leave me alone” kind of attitude, which I can sense. . . . I didn’t realize that she might also be feeling depressed about the situation. I was too concerned about the care that I’m giving her, “Don’t do this. Don’t do that!” etc. Appraising their own caregiving and noting improvements in the way they coped and provided care: I think there is an improvement. Before that I am always “why they think this way? Why my mother-in-law think poor of me this way” but now I will stop. Because I did share [in the group], but someone told me that I can have these feelings, but I must always move one step back and look at the whole situation. Don’t always think that you are right. Shift in worldview e.g. acceptance of uncertainty: I think the challenge, this uncertainty as part of our new life. Because if you see it as a problem, it will always be a problem and it will always be difficult; this is reality. At this point I am still how to incorporate this factor into my life. I keep thinking that my father’s life is going to be uncertain, then how am I going to live my life—it is not like you are waiting for something to happen and then you can be free; it doesn’t work like that. Challenging cognitions as a way of problem-solving: Like I mentioned, I think from the start I was looking at answers. And then when I went through the first session, and the looking at the body language of everyone and the facilitator . . . then I know the gist of the support group is not trying to find the answers. It’s more about providing the listening ear, giving the opportunity, the window to actually speak out things that you don’t speak to anyone else. . . . I think if I were to look for answers instead of trying to understand, or if I not given the opportunity to be given the listening ear, I think things are still the same.
Feedback included content and structure	More homogeneity of cancer types, stages and relationship status within the group preferable: I’d like to see caregivers who’re caregiving for the same person, sessions just for themselves. Like people who are looking after their spouses. I don’t want to be in a group with 5 other people looking after their spouses in palliative care. Because their emotions and reactions to things will be completely different from mine. Importance of the physical venue: If there are too many people there will be too much talk in an hour. The environment was also very good. But it can be better if it were less bright. The space is just enough for the group because I want us to be closer to each other. Longer sessions: I think you all try to kept it to one hour so you all make it available to everybody and also that it is not so taxing for caregivers. But sometimes I find that 1 hour is a bit too short. Like sometimes I find that even if I have things it may not be nice to share for too long or too much because there might not have enough time, I may jump into other people’s time, a bit cautious. More contextualised examples, and more time for caregivers to share with one another: ‘Cos [psychologist] is maybe theory, don’t really involve, real life experiences or real feelings you see. Unlike those caretakers [sic] right, they really feel it, is a real experience, they are taking care of the patient.

## Discussion

Findings from this study suggest that the COPE intervention was particularly helpful in reducing perceived burden. Understandably, QOL domains that are beyond participants’ loci of control, such as disruptiveness and financial concerns, were not amenable to change, and the stability of the domain of positive adaptation may be a result of the short time frame in which the intervention was conducted and evaluated. In addition, participants’ anxiety and depression scores were below subsyndromal cut-offs; the floor effect associated with the HADS may have hampered clear measurement.

However, interviews revealed that participants found the intervention helpful, especially in providing a safe space to ventilate and for social comparisons. Existing literature has described social comparisons as being upward, downward or lateral comparisons with peers, with effects on self-perception, self-evaluation, affect, and behavior [17]. However, because the impact of social comparisons are moderated by personal factors such as levels of self-esteem and perceived control [18], the actual effect among intervention caregivers here may vary across individuals.

Nonetheless, given the setting of an Asian cultural context, support group interventions may be particularly beneficial in addressing the psychosocial needs of caregivers, as compared to individual psychotherapy. Cultural norms may discourage Asian caregivers from seeking external help; there is both a stigma associated with utilizing mental health services [19], as well as a reluctance to discuss personal struggles, such as caregiving difficulties, with non-family members [20]. However, Asian caregivers appear willing to open up and share experiences with other caregivers whom they perceive as being in similar situations [20], making these peer group interventions a suitable strategy to support Asian caregivers.

### Limitations

The non-randomization study design resulted in the creation of non-equivalent groups, as reflected by the higher levels of distress at baseline among the intervention group than the control group. This may have compromised the comparability of both groups and the accuracy in evaluating the effects of the intervention. Nonetheless, we opted for this quasi-experimental design as our priority was to ensure that caregivers had access to the program if they were willing to avail themselves to attend.

### Future recommendations

Recommendations for similar interventions in the future can be gleaned from the experiences of this pilot study. Firstly, while control participants were never available for the intervention, possibly due to hectic schedules, it is likely, and heartening to note, that participants who recognized that they needed help were willing to make time for the intervention, possibly explaining for their poorer emotional wellbeing at baseline. Hence, an enhanced screening of needs, and greater flexibility and individualization of the program may be required, in order to identify and offer timely support for caregivers who are in greatest distress.

Secondly, while the program was provided at no cost to caregivers, which may have enhanced its appeal, it may not be sustainable in the long run. A possible solution could be the integration of such a program into routine service delivery.

Finally, caregivers had expressed interest for additional sessions; the COPE intervention, which combines psychoeducation, skills training, and therapeutic counseling, could be further developed through transferring didactics to an online platform, with time dedicated to clarifying doubts and supportive expressive therapy.

While further research is essential in replicating and expanding upon the findings of this pilot service in an Asian setting, the hope is that such a program could eventually be effective in Singapore in supplanting sessions with mental health professionals for family caregivers of cancer outpatients.

## Abbreviations

COPE: Caregivers of cancer Outpatients’ Psycho-Education support group therapy
CQOLC: Caregiver Quality of Life Scale–Cancer
HADS: Hospital Anxiety and Depression Scale
NCIS: National University Cancer Institute, Singapore
QOL: Quality of life
